# Land use drives differential resource selection by African elephants in the Greater Mara Ecosystem, Kenya

**DOI:** 10.1186/s40462-023-00436-8

**Published:** 2024-02-01

**Authors:** Jake Wall, Nathan Hahn, Sarah Carroll, Stephen Mwiu, Marc Goss, Wilson Sairowua, Kate Tiedeman, Sospeter Kiambi, Patrick Omondi, Iain Douglas-Hamilton, George Wittemyer

**Affiliations:** 1 Mara Elephant Project, Nairobi, Kenya; 2 Colorado State University, Fort Collins, USA; 3 Kenya Wildlife Research and Training Institute, Naivasha, Kenya; 4 Max Planck Institute of Animal Behavior, Constance, Germany; 5 Save the Elephants, Nairobi, Kenya

**Keywords:** GPS tracking, Home-range, Resource selection, Bayesian, Loxodonta africana, EarthRanger, Ecoscope, INLA, Landscape dynamics, Land cover

## Abstract

Understanding drivers of space use by African elephants is critical to their conservation and management, particularly given their large home-ranges, extensive resource requirements, ecological role as ecosystem engineers, involvement in human-elephant conflict and as a target species for ivory poaching. In this study we investigated resource selection by elephants inhabiting the Greater Mara Ecosystem in Southwestern Kenya in relation to three distinct but spatially contiguous management zones: (i) the government protected Maasai Mara National Reserve (ii) community-owned wildlife conservancies, and (iii) elephant range outside any formal wildlife protected area. We combined GPS tracking data from 49 elephants with spatial covariate information to compare elephant selection across these management zones using a hierarchical Bayesian framework, providing insight regarding how human activities structure elephant spatial behavior. We also contrasted differences in selection by zone across several data strata: sex, season and time-of-day. Our results showed that the strongest selection by elephants was for closed-canopy forest and the strongest avoidance was for open-cover, but that selection behavior varied significantly by management zone and selection for cover was accentuated in human-dominated areas. When contrasting selection parameters according to strata, variability in selection parameter values reduced along a protection gradient whereby elephants tended to behave more similarly (limited plasticity) in the human dominated, unprotected zone and more variably (greater plasticity) in the protected reserve. However, avoidance of slope was consistent across all zones. Differences in selection behavior was greatest between sexes, followed by time-of-day, then management zone and finally season (where seasonal selection showed the least differentiation of the contrasts assessed). By contrasting selection coefficients across strata, our analysis quantifies behavioural switching related to human presence and impact displayed by a cognitively advanced megaherbivore. Our study broadens the knowledge base about the movement ecology of African elephants and builds our capacity for both management and conservation.

**Supplementary Information:**

The online version contains supplementary material available at 10.1186/s40462-023-00436-8.

## Introduction

Many wildlife areas are undergoing rapid anthropogenic land conversion resulting in habitat loss and fragmentation that continue to be leading causes of species extinctions and biodiversity loss globally [1–3]. Effective habitat management and conservation planning requires a comprehensive evidence-based understanding of species-environment relationships [4]. In areas of anthropogenic-driven land use conversion, species’ behavior often differs across contiguous land parcels [5], which can be critical for persistence. Conservation in complex, variegated landscapes is facilitated by building a deeper understanding of the drivers of, and changes in, habitat selection – the behavioral process that occurs across a hierarchy of spatiotemporal scales, in which animals actively or passively choose habitats to fulfill requirements for survival, growth, and reproduction while balancing fitness and predation risk [6–9]. Quantifying changes in habitat selection across contiguous areas with different human impacts can serve to identify landscape features relied upon for persistence in different contexts that may be a target for management efforts.

Habitat selection is fundamentally driven by trade-offs between food access and perceived mortality risk, which can change dramatically in landscapes at the human-wildland interface [10]. For species that are harvested or persecuted by humans, perceived mortality risk (e.g., ‘the landscape of fear’; [11]) and consequently habitat selection is often shaped by the presence of human infrastructure and population centers [12–14] and human activities like hunting [15] or agriculture [16]. Perceived risk from humans may also vary temporally depending on the timing of human activity (e.g., less risk at night) [17] or may be tolerated differently by individuals in a population based on factors such as sex [18].

Resource Selection Functions (hereafter RSFs; [19]) are commonly used to quantify animal habitat selection. Typically, RSF models are structured with a use-versus-availability design that compares environmental data at species occurrence or use points (i.e., GPS relocation data) to environmental data at a sample of points assumed to be available but not observed [20]. Such models provide estimates that are proportional to the probability of use for a given habitat unit [21, 22]. RSFs are useful tools for landscape planning and habitat management because they can be used to make spatially explicit inference [20]. Increasingly, RSFs are applied to assess anthropogenic effects on space-use and movements of wildlife [23–25]. For example, RSF model estimates have been used to map species distributions and predict range shifts [4, 26], model functional connectivity [27, 28], and quantify animal responses to anthropogenic land use change [12, 29].

Resource selection behavior of individual animals can change as a function of resource availability (e.g., a functional response; [30]). Characterizing animal functional responses in resource selection is increasingly of interest to improve understanding of how resource selection behavior changes across ecoregions [25] or across gradients of human activity or disturbance [31–33]. For example, [31] evaluated wolf resource selection as a function of proximity to humans and found that in areas with high human activity, wolves selected locations closer to humans but avoided human activity during daylight hours, whereas in areas with low human activity, proximity to humans did not influence wolf resource selection [31]. However, few studies have investigated how species might shift (intensify or weaken) or flip (selection to avoidance) resource selection between discrete land management areas within their home range (but see [34, 35]). Understanding how specific land management regimes affect wildlife resource selection, particularly for wide-ranging, persecuted species with large space-use requirements, is key to informing optimal conservation and management actions.

African savanna elephants (*Loxodonta africana*) are an ideal species for studying the effects of land management on selection because they have large home ranges, high dispersal potential, and are habitat generalists known to use space outside of protected area boundaries [36, 37]. In addition, elephants are persecuted by humans for ivory and their involvement in human-wildlife conflict. In 2021, the International Union for Conservation of Nature (IUCN) updated the status of African savanna elephants to “Endangered” under the IUCN Red List Assessment [38]. Across elephant range countries, previous studies have identified human presence [25, 39], land fragmentation [5, 40], water availability [25, 41], vegetation productivity and structure [25, 35, 42], and slope [43] as factors that influence elephant resource selection, but less is known about how selection for these features changes in relation to human land use management practice. In particular, community-based conservation, where land management is intended to support both wildlife conservation and human social wellbeing through development initiatives and land-sharing, has been widely adopted across sub-Saharan Africa in elephant range countries over the last twenty years [44, 45]. Yet, little information about the impact of community-based conservation on African elephant resource selection and space use is available, and no studies have directly contrasted variation in African elephant resource selection within home ranges between community-based conservation areas, conventional formally protected areas, and unprotected areas.

In this study, we use GPS telemetry data of African elephants in the Greater Mara Ecosystem of Kenya to develop third-order (i.e., within home range) resource selection functions and investigate how resource selection by elephants shifts across land management zones that correspond to discrete differences in human land-use intensity and protection for elephants [9]. Our main objectives were to (1) understand spatial variation in selection across management zones, and (2) explore diurnal, sex and seasonal differences in selection across this spatial structure [10, 35, 46]. We expected that (1) differences in selection would occur primarily in relation to anthropogenic features (i.e. greatest avoidance in unprotected areas and greater selection for cover habitat in unprotected areas), (2) similarities in selection would occur in relation to geophysical and foraging resources (i.e., avoidance of slope and attraction to vegetation productivity) across management zones, and; (3) sex, diurnal and seasonal differences in selection would be greater in unprotected areas (where human activities have stronger influence). We discuss how these results can facilitate understanding of elephant behavioral adaptations to human activity and provide insight to conservation efforts in ecosystems with variable land use management systems.

## Methods

### Study area

**Fig. 1:**
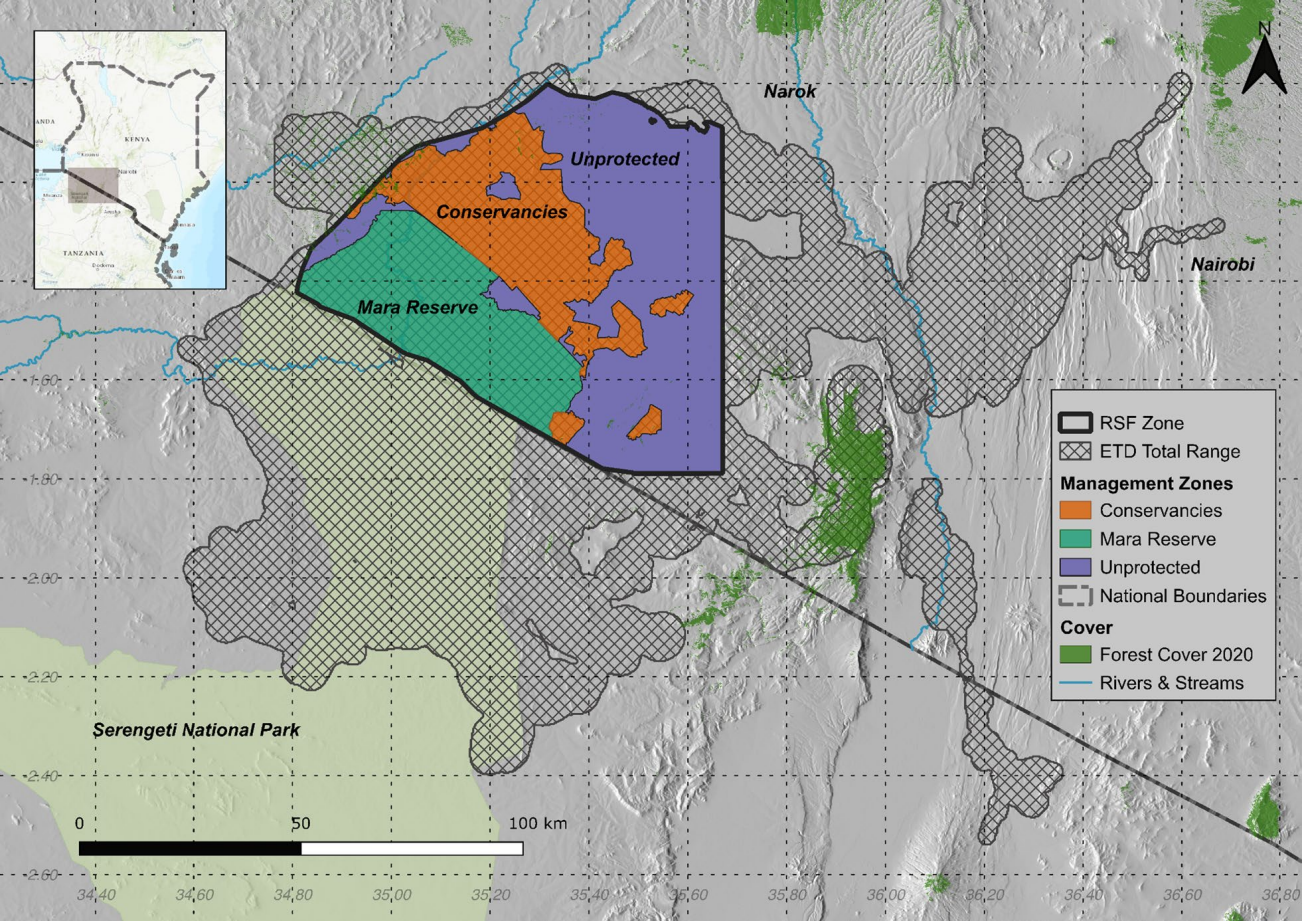
The combined 100th percentile Elliptical Time Density (ETD) Home Range Area from 58 elephants (18,795 sq.km) between 2011 and 2022 (grey hatching). The combined Mara Reserve (green), Conservancies (brown), and Unprotected (purple) zones form the Resource Selection Function (RSF) Zone (black outline) where the RSF analysis was conducted based on the union of spatial layers and tracking data. Within the RSF Zone were 834,138 used positions and 9,180,738 unused positions from 49 elephants

The study area of 5,709 km^2^ is situated within the Greater Mara Ecosystem (GME) – the northern extent of the transboundary Serengeti-Mara Ecosystem (Fig. 1). The core area of the GME is formed by the 1,500 km^2^ Maasai Mara National Reserve, where photo tourism is the only permitted land use. This core area is buffered by community conservancies covering 1394 km^2^, which allow regulated use of the landscape for livestock grazing in addition to tourism [47, 48]. The remainder of the GME is unprotected and made up of private land parcels with human settlement, agriculture and pastoralism. These areas remain critical to dispersing and migrating wildlife and incur high rates of human-wildlife conflict [49, 50].

Seasonal rainfall patterns are generally bimodal, with two wet periods and two dry periods annually but rainfall is becoming more variable overall [51, 52]. Annual rainfall follows a spatial gradient from 600 mm in the southeast to 1300 mm in the northwest Transmara region [52, 53]. Rainfall patterns structure wildlife distributions throughout the year, and long-distance movements are common to access areas of higher vegetation productivity. The GME is tropical grassland savanna interspersed with Acacia woodlands but includes dense bushland thickets and some montane forest.

Recent estimates put the elephant population in the Serengeti-Mara Ecosystem at 7535 individuals with 2595 within the GME [54]. Elephants in the GME remain threatened by human elephant conflict, illegal hunting for ivory, expansion of agriculture, and a surge of fencing, road development, and settlements in unprotected corridors and dispersal areas [50, 55, 56]. Given the rapid changes occurring across the landscape in relation to increasing human pressures, identifying and characterizing high value features on the landscape for elephants is important for informing the ecosystem management process currently underway.

### Tracking data

Fifty-eight elephants were collared between September 2011 and April 2022 across the GME. GPS collar deployments followed four objectives: (1) elephants with a high poaching risk (e.g., large tusked individuals), (2) conflict risk (e.g., individuals known to move through high settlement density areas), (3) elephants thought to be presenting interesting corridor or long-distance movements, and; (4) to collect a balanced spatial representation of the Mara ecosystem. Females selected for collaring each represented a distinct family group, while selected males were dispersed at the time of collaring. Elephants were immobilized and fitted with GPS collars following procedures established by the Kenya Wildlife Service. The study used collars from African Wildlife Tracking™, Savannah Tracking™, Vectronics™, and Followit™. Collars were most commonly set to collect GPS points every hour and data were transmitted by satellite or cellular signal. During capture, ages were estimated when possible based on body size and molar progression [57].

We evaluated resource selection by elephants using third-order RSFs based on a use-available design [20, 21]. RSFs rely on comparing GPS locations used by an animal to random locations on the landscape that are considered to be available to each individual but not necessarily used. To characterize availability, we constructed 100th percentile elliptical time-density (ETD) home ranges [58] for each individual and generated 10 random unused locations within each individual’s home range for every used GPS location [59]. For every unused sample we generated a random timestamp between the data start and end time for a given individual. We classified the set of used and available GPS positions into one of three land management zones: Maasai Mara National Reserve (*Mara Reserve*), community conservancy areas (*Conservancies*), and unprotected areas (*Unprotected*).

### Covariate data

Covariates were classified as either (i) geophysical (*Slope, Drains, Bare)*, (ii) vegetation (Normalized Difference Vegetation Index (*NDVI*) and *Cover*), and; (iii) anthropogenic (*Agriculture, Lodges, Settlements, Roads*). *Slope* was calculated from NASA’s Shuttle Radar Topography Mission at 30 m resolution [60]. Moderate Resolution Imaging Spectroradiometer (MODIS) Aqua/Terra 16-day 500 m composite normalized difference vegetation index (*NDVI*) images [61] were used to annotate each used/unused observation with the closest spatial and temporal NDVI value within the study period (2011–2022). To capture water availability, we included rivers and drainages from the global HydroSHED Free Flowing Rivers Network [62] buffered by 400 m corresponding to the 95th percentile of elephant step length, which served to demarcate the area in which water was readily accessible (*Drains*). We created a land cover map of the Mara using Sentinel-1 and Sentinel-2 imagery from 2019 to 2022 at 10-m resolution, given that currently published land cover maps in the area lacked information on up-to-date agricultural boundaries [63]. Land cover classes were defined by the percent of canopy cover and were defined as < 20% canopy cover (e.g. grassland and savanna and composed 29.8% of study area), 20–70% cover (e.g. open woodland and bushland and composed 46.9% of study area), > 70% cover (e.g. forest and bushland thicket and composed 9.6% of study area), and agriculture (composed 9.7% of study area). Bare ground, rock, and built land were combined into a single class (*Bare*) (composed 1.6% of study area). Land cover classification was performed using random forest classification with 2,696 training points and validated using five-fold cross validation (mean accuracy 81%; Additional file 1: SM Table 2). Seasons (i.e., wet versus dry periods) were delineated using *NDVI* values for the study area using 16-day MODIS imagery. Values were classified into *Wet* and *Dry* periods using gaussian mixture clustering, where high *NDVI* values correspond to wet periods and low *NDVI* corresponds to dry periods [35]. To capture diurnal information, we annotated each used/unused observation with a ‘day/night’ attribute by recording whether the observation’s timestamp was after or before sunrise/sunset. We also quantified human footprint dynamics using digitized permanent settlements and buildings (*Settlements*), tourist lodges and camps (*Lodges*), and ground-mapped primary and secondary roads (*Roads*), each buffered by 400 m to create presence/absence raster layers.

### Data modeling

To answer our research questions, we developed four sets of discrete statistical models consisting of 21 models in total as follows. First, we defined 3 Zonal models (Table 1) to assess generalized selection across the three different management zones within the study site: (i) *Mara Reserve*, (ii) *Conservancies* and iii) *Unprotected*. All model parameters were included within each of the three models except for the inclusion of an agricultural land cover variable in the *Unprotected* model and the lack of a settlement covariate in the *Mara Reserve* model (Table 1). We then defined 6 models each to compare selection in each of the three management zones across strata: season (*Wet/Dry* by zone), time-of-day (*Day/Night* by zone), and sex (*Male/Female* by zone).

**Table 1:** Zonal model definitions

Model structure	Management zone
log($\frac{\theta }{1-\theta }$) = $\beta$_0_ + $\beta$_SLOPE_ + $\beta$_DRAINs_ + $\beta$_NDVI_ + $\beta$_COV-20_ + $\beta$_COV-2070_ + $\beta$_COV-70_ + $\beta$_BARE_ + $\beta$_ROADS_ + $\beta$_LODGES_	*Mara Reserve*
log($\frac{\theta }{1-\theta }$) = $\beta$_0_ + $\beta$_SLOPE_ + $\beta$_DRAINS_ + $\beta$_NDVI_ + $\beta$_COV-20_ + $\beta$_COV-2070_ + $\beta$_COV-70_ + $\beta$_BARE_ + $\beta$_ROADS_ + $\beta$_LODGES_ + $\beta$_SETTLEMENTS_	*Conservancies*
log($\frac{\theta }{1-\theta }$) = $\beta$_0_ + $\beta$_SLOPE_ + $\beta$_DRAINS_ + $\beta$_NDVI_ + $\beta$_COV-20_ + $\beta$_COV-2070_ + $\beta$_COV-70_ + $\beta$_BARE_ + $\beta$_ROADS_ + $\beta$_LODGES_ + $\beta$_SETTLEMENTS_ + $\beta$_AGRICULTURE_	*Unprotected*

The Mara Reserve did not contain an appreciable number of settlements or any agriculture and these covariates were not included in the model definition. Similarly, there is very little agriculture in the community conservancies and this covariate was not included in the model definition. Models for the three comparison strata (season, sex, time-of day) followed the same model structure

We fit all 21 elephant resource selection models using a Bayesian framework. We used a hierarchical model structure to account for inter-elephant differences by allowing individual-level parameter mean and variance values to be drawn from Gaussian group-level parameters. Group-level parameter mean and variances were also assumed Gaussian. Uninformative prior values for group-level mean = 0 and variance = 100 were used for all model parameters. A logistic-link (i.e., sigmoid) function was used to link a linear predictor with a Bernoulli probability $\theta$ (Table 1).

Continuous variables (*Slope & NDVI*) were first normalized to the range 0–1 and then z-score standardized [64]. From fitted models, posterior distributions and 95% highest-density posterior intervals (HDPIs) were calculated for each parameter in the model. To assess the similarity of selection between individual elephants, we visualized the posterior predictive distribution of the individual random effects for each zonal model and assessed the variance. For model diagnostics, we used posterior predictive checks to assess how well the predicted values from each zonal model described the observed data [65].

To understand which data strata (*Season, Time of Day, or Sex*) most strongly structured resource selection behavior, we used a ‘consistency’ score [66] calculated as the mean of the absolute difference between zonal selection coefficients *i ∈ {Mara Reserve, Conservancies, Unprotected}* for a strata level *j ∈ {Zone, Season, TOD, sex}* for a given covariate *k ∈ {1…n}*. The consistency score ${C}_{ijk}$ can be aggregated across combinations of indices *i, j, k,* and is always positive with values further from zero indicating more differentiation. For example, the consistency of resource selection parameters *p* across each zone *i* = *{Mara Reserve (mr), Conservancies (cca), Unprotected (up)}* for strata *j* = *’sex’* within covariate *k* = *’NDVI’* would be calculated as:

$${C}_{ijk}= \frac{\left(\begin{array}{c} \left|{p}_{{i}_{mr}{j}_{f}{k}_{ndvi}}-{ p}_{{i}_{cca}{j}_{f}{k}_{ndvi}}\right| +\left|{p}_{{i}_{mr}{j}_{f}{k}_{ndvi}}-{ p}_{{i}_{up}{j}_{f}{k}_{ndvi}}\right| + \left|{p}_{{i}_{cca}{j}_{f}{k}_{ndvi}}-{ p}_{{i}_{up}{j}_{f}{k}_{ndvi}}\right| + \\ \left|{p}_{{i}_{mr}{j}_{m}{k}_{ndvi}}-{ p}_{{i}_{cca}{j}_{m}{k}_{ndvi}}\right| +\left|{p}_{{i}_{mr}{j}_{m}{k}_{ndvi}}-{ p}_{{i}_{up}{j}_{m}{k}_{ndvi}}\right| + \left|{p}_{{i}_{cca}{j}_{m}{k}_{ndvi}}-{ p}_{{i}_{up}{j}_{m}{k}_{ndvi}}\right| \end{array}\right)}{6}$$

We used consistency to address objectives related to determining the relative differentiation in resource selection behaviour between management zones and within management zones with respect to diel timing, season, and sex.

### Data & analytical processing

Elephant tracking data were collected, stored and accessed using the *EarthRanger* platform (www.earthranger.com). Spatial vector covariate layers were stored and accessed from the *Landscape Dynamics* spatial database [67]. Raster-based covariate layers were stored and accessed from the *Google Earth Engine* platform [68]. All analytical and data processing steps were performed using the open-source *Ecoscope* python library (https://ecoscope.io) within *Jupyter Notebooks* (https://jupyter.org). All Bayesian statistical models were fit using the *INLA* v22.10.23 [69] package for *R* (www.r-inla.org). We developed a *Terraform* (https://www.terraform.io/) server definition to quickly deploy and run our computational infrastructure on the *Google Cloud Platform* (https://cloud.google.com/) using an *n1-highmem-32* (https://cloud.google.com/compute/docs/general-purpose-machines#n1-high-memory) machine in order to process the entire 9.18 M used/unused observations in the dataset. We configured our analysis environment with *Docker* (https://www.docker.com/) to support both necessary *Python* and *R* kernels and analytical replicability. All *Jupyter Notebooks*, *Terraform* and *Docker* files needed to reproduce our analyses are available in the supplementary info (Additional file 2).

## Results

Fifty-eight elephants were tracked from Sept 2011–April 2022 totalling 1,176,287 positions (interquartile range: 8844–29,991). The combined total Elliptical Time Density (ETD) elephant home-range covers an area of 18,758 sq.km extending west from the Mara reserve into Nyakweri forest, south into the Serengeti and Loliondo in Tanzania, and east to the Loita forest and extending through the Great Rift Valley (Fig. 1). Of the elephants tracked 44 had datasets greater than one-year in duration which we used to report Elliptical Time-Density (ETD) [36] individual range metrics. The 100th percentile home range areas of these 44 individuals averaged 1455 sq.km (Interquartile Range: 892–1700 sq.km). Wet season ranges (mean = 1098 sq.km, IQR: 606–1336 sq. km) were similar but slightly smaller than dry season ranges (mean = 1192 sq.km, IQR: 745–1623 sq. km). There was on average 56% overlap between individual wet and dry season range areas (IQR: 47%–66%).

We retained 49 elephants and 834,138 GPS locations for the RSF analysis that intersected with the extent of the covariate layers. This subset of data contained 26 male (416,215 locations) and 23 female elephants (417,923 locations). 17% of locations were recorded within the MMNR (15 female / 101,938 locations, 19 males / 42,151 locations), 59% in community conservancies (18 female / 228,556 locations, 24 male / 264,950 locations), and 24% outside formally protected lands (20 female / 87,429 locations, 24 males / 109,114 locations).

**Fig. 2:**
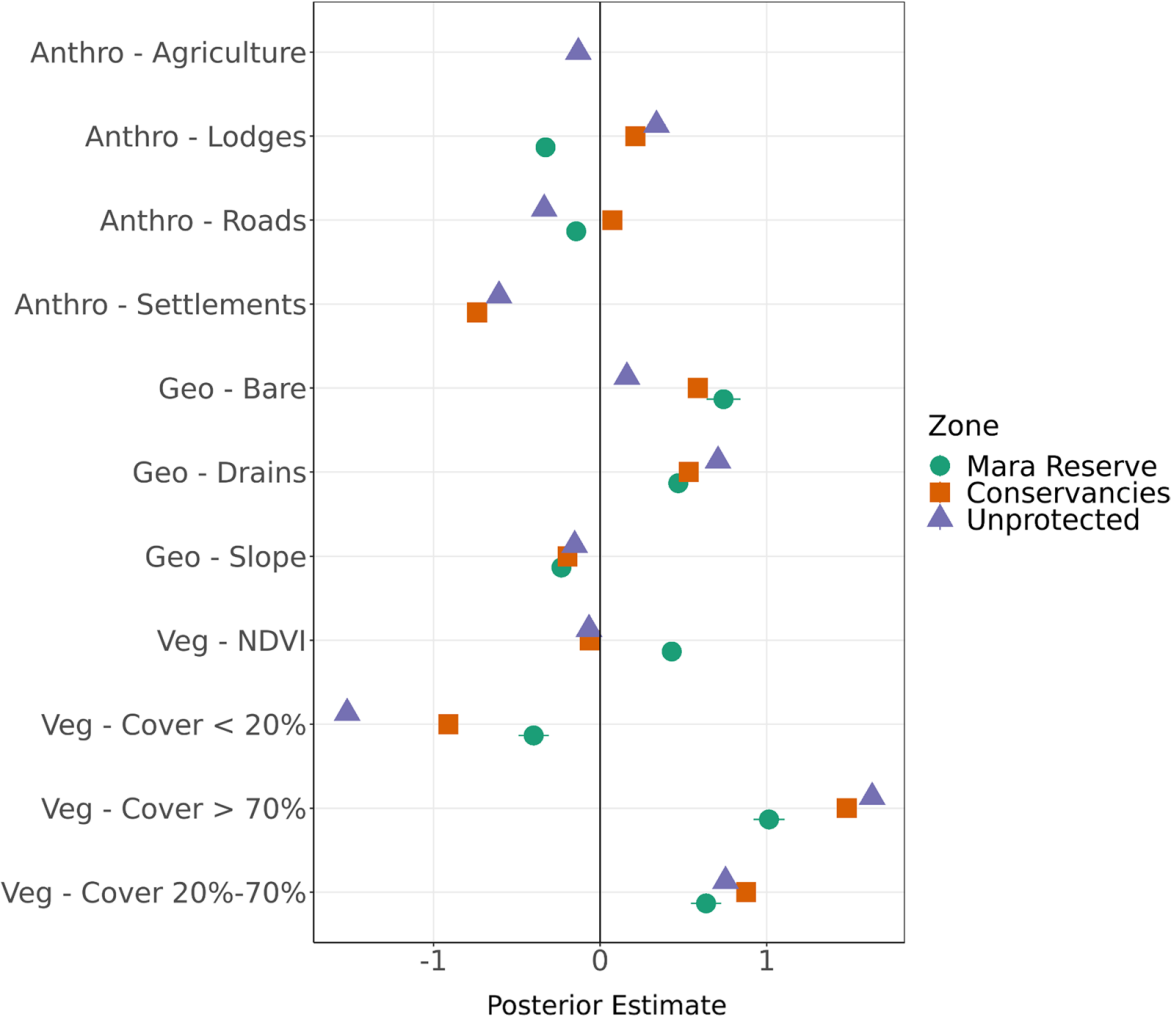
Parameter mean and 95% Highest Posterior Density Interval (HPDI) estimates for each spatial covariate within the three management zone models: i) Mara Reserve, ii) Conservancies, iii) Unprotected zones

All 21 models converged and posterior predictive checks showed that all models accurately described the observed data (Additional file 1: SM Fig. 2). Results from the zone-only strata models, contrasting selection in areas with different human land use and management, showed elephant selection behavior differed significantly across zones, particularly in respect to vegetative (*NDVI, Cover* < *20%. Cover 20%-70%, and Cover* > *%70*) and anthropogenic (*Agriculture, Lodges, Roads, Settlements*) features (Fig. 2, Additional file 1: SM Table 1). In contrast, selection coefficients for geophysical (*Bare, Drains and Slope*) features were more similar across zones (Fig. 2, Additional file 1: SM Table 1). Selection in respect to vegetation *Cover* demonstrated universal positive selection for *Cover* between 20% and 70%, for *Cover* over 70% and for *Bare* across management zones (Fig. 2, Additional file 1: SM Table 1). Coefficient values were generally stronger in the *Conservancies* and *Unprotected* areas, with the highest values (strongest selection) for vegetation *Cover* over 70% (mean: 1.634). In contrast, vegetation *Cover* under 20% had negative selection across all regions, with stronger avoidance in *Unprotected* (mean: -1.519) and *Conservancies* (mean: -0.912) compared to the *Mara Reserve* (mean: -0.399) (Fig. 2, Additional file 1: SM Table 1). Contrary to our prediction, *NDVI* had both a small selection magnitude and the least variation in selection of any vegetative covariate between land use areas, with negative selection in *Unprotected* (-0.067) and *Conservancies* (-0.061) but positive selection in *Mara Reserve* (0.430)*.* Selection of anthropogenic features differed by zone and feature: *Settlements* were avoided in *Conservancies* (-0.738) and *Unprotected* (-0.067) and were not present in the *Mara Reserve*. There was negative selection for *Lodges* (-0.328) in the *Mara Reserve* but they were selected for in the *Conservancies* (0.211) and *Unprotected* (0.338). *Roads* were avoided in *Unprotected* (-0.336) and *Mara Reserve* (-0.144) but had very slight positive selection in the *Conservancies* (0.074). *Agriculture* was avoided in *Unprotected* (-0.131) (and not present in *Conservancies* or *Mara Reserve*). Selection for geophysical features was universally negative for increasing slope, particularly in the *Mara Reserve* (-0.232) and *Conservancies* (-0.196), and universally positive for *Drains*, particularly in *Unprotected* (0.708) and *Conservancies* (0.531) (Fig. 2, Additional file 1: SM Table 1)*.*

Assessment of the group-level posterior distribution of the individual random effects for each zonal model showed that the most variation between individuals occurred in the *Mara Reserve*, while variation was the lowest in *Conservancies* (Additional file 1: SM Fig. 1a-b). The *Unprotected* had several individuals with differing posterior distributions, but the majority of individuals were clustered (Additional file 1: SM Fig. 1c).

**Fig. 3:**
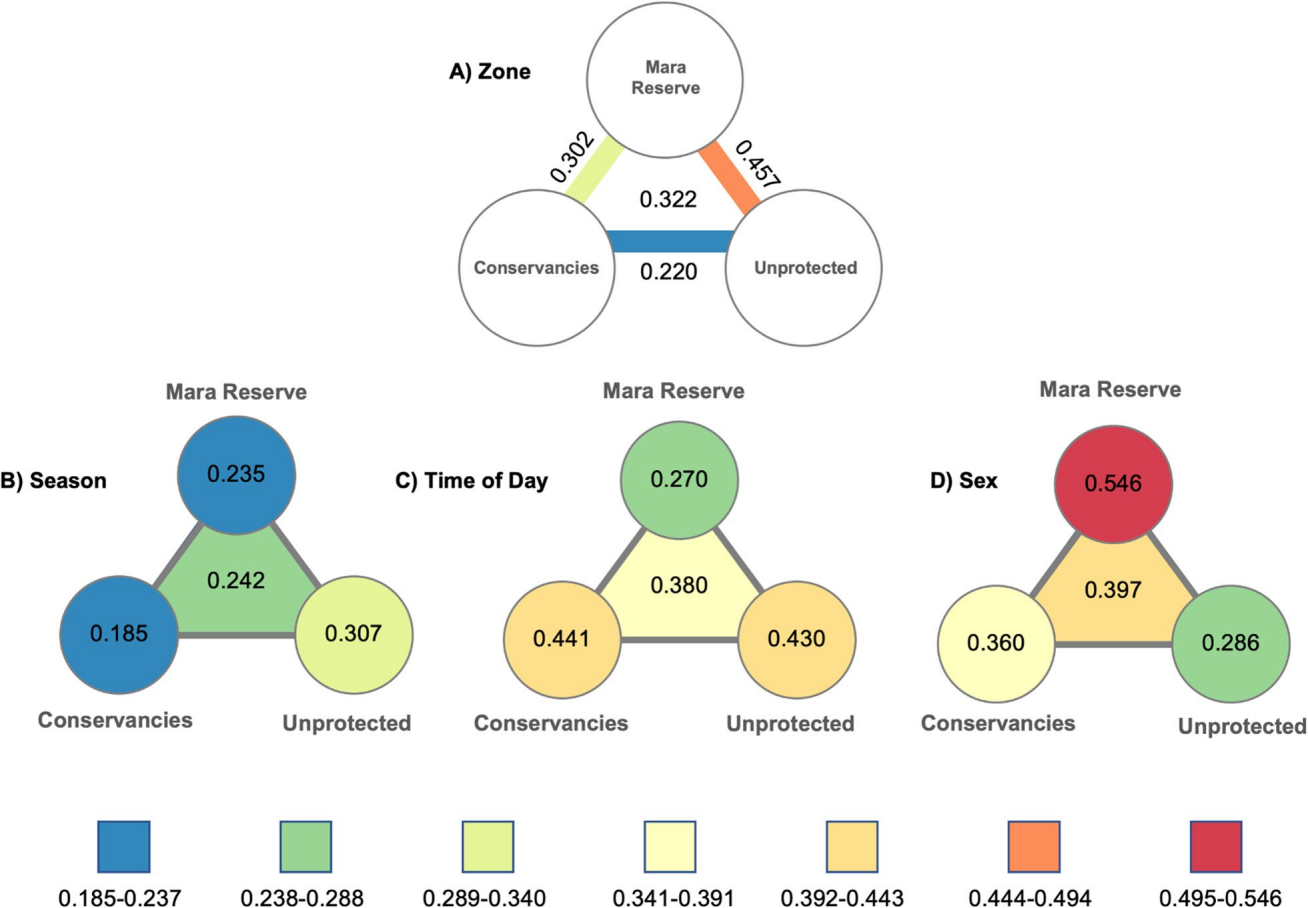
Consistency scores are shown for each data contrast (strata). A higher consistency score indicates a higher differentiation of parameter values across the comparison strata. Values have been colour coded blue (low differentiation) through red (high differentiation). Consistency metrics were calculated between zones (values between circles) for the Zone models **A**, and across zones (center values) for Season **B**, Time of Day **C**, and Sex **D**

**Fig. 4:**
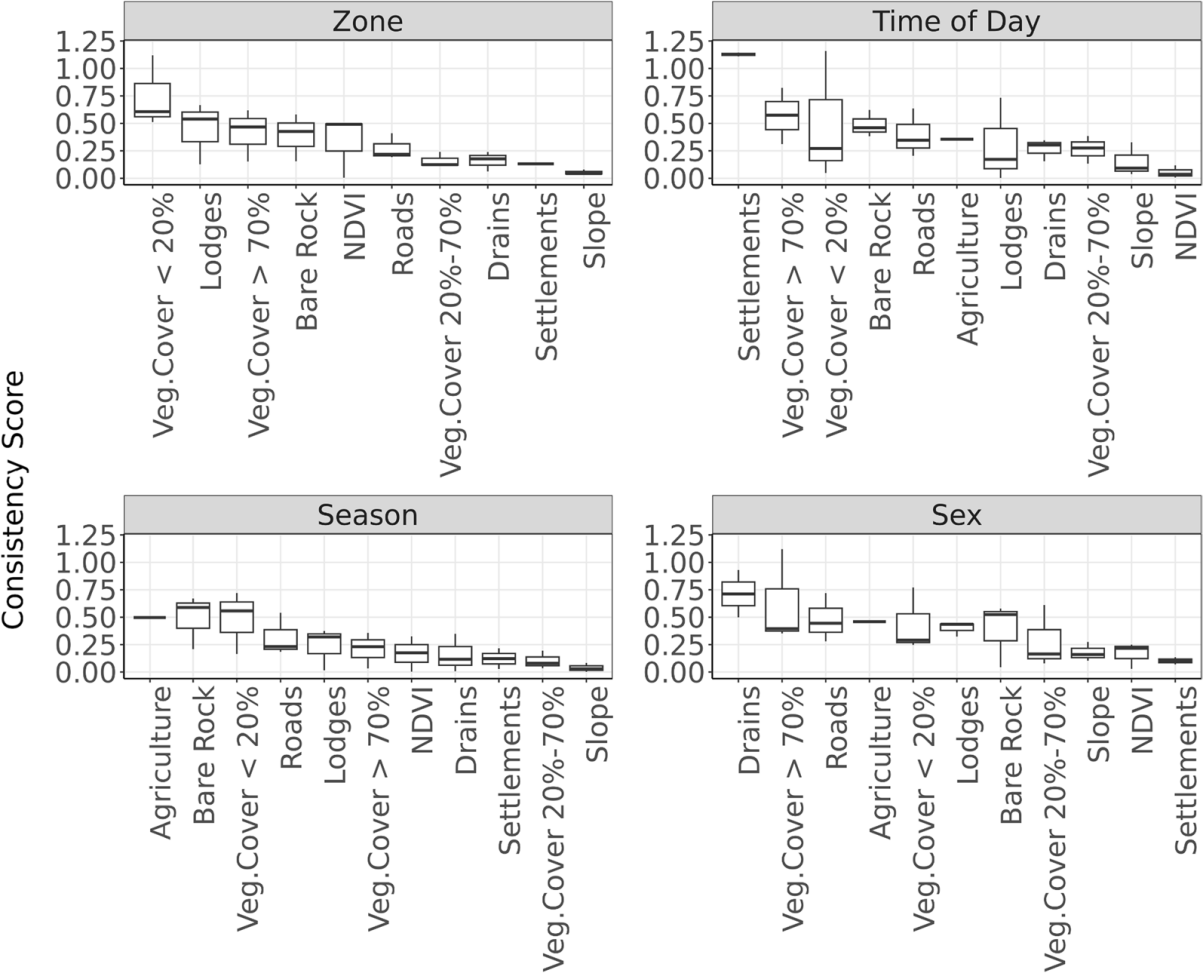
Consistency scores summarised for each strata ${{\varvec{C}}}_{{\varvec{n}}}$. The x-axis shows the model covariates. The y-axis (Consistency Score) represents the mean of the absolute differences of covariate parameter estimates across the three land management zones and across each strata level for a given covariate. The strata are Zone, Time of Day, Season, and Sex

Differentiation in selection coefficient values (consistency) was strongly related to the gradient of protection between management strata when analyzing the data according to zone only. The greatest difference occurred between the *Unprotected* and *Mara Reserve* ($C=0.457$, Fig. 3A). Difference in selection was lowest between the *Conservancies* and *Unprotected* ($C=0.220$, Fig. 3A), while *Mara Reserve-Conservancies* differences fell in the middle ($C=0.302$, Fig. 3A). The overall difference value across all land uses with the zonal model was $C=0.322$ (Fig. 3A). Degree of avoidance of cover below 20% was the main driver of differentiation across zones, with stronger avoidance in the *Unprotected* and *Conservancies*, and avoidance of slope showed the strongest consistency across zones (Fig. 4A).

Possibly in relation to the relatively high degree of overlap between seasonal home ranges, seasonal model coefficient values were the most similar ($C=0.242$, Fig. 3B) of any strata comparison, suggesting selection behavior did not shift strongly with respect to season in the study system. Seasonal differentiation was lowest within *Conservancies* ($C=0.185$, Fig. 3B) and greatest in *Unprotected* ($C=0.307$, Fig. 3B) with intermediate differentiation in *Mara Reserve* ($C=0.235$, Fig. 3B). The main covariate driving differentiation was avoidance of agriculture (closely followed by bare ground and cover below 20%), while avoidance of slope had strong consistency (Fig. 4C).

We found much stronger diurnal differentiation in selection than that noted between seasons, with an overall difference index of 0.380 (Fig. 3D). This value suggests there was more differentiation in selection behavior between night and day hours than between wet and dry seasons or across management zones. Diurnal consistency scores were similar between the *Conservancies* (0.441, Fig. 3D) and *Unprotected* (0.430, Fig. 3D) indicating strong diurnal structuring of selection. The *Mara Reserve* had notably less differentiation between day and night selection (0.270, Fig. 3D), where differentiation tended to be less than that found across management zones. Avoidance of settlement was the strongest driver of differentiation, while NDVI had the least differentiation (Fig. 4B).

Intra-strata comparisons found that the greatest degree of differentiation in resource selection occurred between sexes with an overall consistency of $C=0.397$ (Fig. 3C) – a value slightly greater than that found in the diurnal and management zone contrasts. The greatest sex-based differences occurred in the *Mara Reserve* zone ($C=0.546$, Fig. 3C), being the highest consistency score across any strata in any zone in the study. Differences between coefficient values were notably lower in the *Conservancies* ($C=0.360$, Fig. 3C) and *Unprotected* ($C=0.286$, Fig. 3C), demonstrating sex-based differences in selection behavior were less pronounced in areas experiencing greater human activity. Selection for drainages drove differentiation across zones, while avoidance of settlement was the most consistent (Fig. 4D).

## Discussion

African elephants are intrinsically linked to many emergent conservation issues [70] including human-wildlife conflict [71], climate change [72], range fragmentation [40] and consumptive poaching and hunting [73, 74]. Elephants have large home-ranges and requirements for vegetative and water resources which puts them at risk from the expanding human-footprint across most of their continental range [36]. The Greater Mara Ecosystem is an area undergoing rapid human-footprint expansion including fencing [55, 75, 76], agricultural expansion and industrialization [77], livestock increase [50], deforestation [78], accelerated human population growth [50, 79], and wildlife declines [50]. It is also still considered to be a globally premiere wildlife and biodiversity hotspot and represents important economic revenue for Narok County and Kenya as a whole [80]. The landscape is complex, including governmentally protected areas, community conservancies, and unprotected agricultural and range lands, all of which are transboundary with Tanzania. These diverse factors make the study of the distribution and movement of elephants in the Mara, especially with regards to management and anthropogenically influenced factors, particularly important. This analysis, using over ten years of intensive tracking data, represents the first comprehensive definition of range and resource use by elephants in the ecosystem.

In this study, we evaluated elephant range and assessed resource selection behavior across management zones (objective 1), and in relation to season, time of day and sex (objective 2) using third-order selection (i.e., selection/avoidance by individuals within each elephant’s home-range). We looked at both the relative magnitude and sign (positive/negative) of selection coefficients and at the differences in coefficients across strata.

### Resource selection across zones

Vegetative cover had the strongest influence on elephant selection with cover > 70% being the most strongly selected covariate and cover < 20% being the largest magnitude negative selection covariate. Vegetative cover > 70% was most strongly selected for in the *Unprotected* and *Conservancies* zones, while cover < 20% was most strongly avoided in the *Unprotected* and *Conservancies* and supporting hypothesis (1). This points to the importance of forest cover as habitat in human-dominated areas, likely representing a response to the ‘landscape of fear’ [11]. Several studies have reported on the relationship between elephants and NDVI and elephants are generally believed to favour areas with greener vegetation [81] especially in more arid landscapes [35, 82]. Our results show however that in the GME, there is only a relatively weak selection for higher NDVI, and even negative selection outside of the formally protected wildlife area. In our analysis region, because of extensive grass cover, there was some correlation between NDVI and cover > 70% (0.38) (Additional file 1: SM Fig. 6) which may temper the overall NDVI signal. However, [36] also found that when considering overall landscape effects, NDVI was not an important driver relative to anthropogenic influence and only became important over localized scales. This difference in the role of NDVI in structuring elephant space use is likely related to the high overall productivity in the Mara, which is on the wetter side of the mesic savanna ecotone.

### Key differences across RSF models

Our results indicated that selection behavior differed most strongly by sex followed by time of day, then by management zone, and finally by season. For each of these contrasts, the magnitude of differences in selection behaviour appeared to be structured by human activities. For instance, differences in selection between sexes were most pronounced in the Mara Reserve, and much weaker in human dominated landscapes, likely reflecting limitations in possible resource use strategies driving more similarity between sexes in human dominated areas. Similarly, the strong diurnal differences in resource selection behavior in the study ecosystem was likely a response to highly diurnal human activity [83, 84], being greatest in areas with high human activity. Overall, resource selection was more similar in the mixed wildlife-livestock *Conservancies* and *Unprotected* zones relative to the wildlife-only zone of the Mara reserve, suggesting less scope for differentiation in resource selection behavior when in human dominated areas (supporting hypothesis 3). Selection for open grassland and high canopy cover areas appeared to be key drivers of this differentiation across strata, with elephants selecting for high canopy cover and avoiding open areas most strongly in unprotected areas. This highlights the universal importance of high canopy cover in human dominated areas for elephants and consequently the importance for protection of forested areas.

Across management zones, the strongest driver of differentiation in resource selection was avoidance of cover < 20%. Vegetation cover < 20% had the largest gradient change in selection magnitude of any covariate, where elephants strongly avoided open-areas within the *Unprotected* region, but avoidance was weaker in the *Conservancies* and weakest in the *Mara Reserve*. In the study system, cover < 20% is primarily open grasslands that are used extensively by grazers, including livestock, especially within the *Conservancies* and *Unprotected* regions. As such, it is plausible that elephants were avoiding livestock and herders when exposed to them in this land cover category in the *Conservancies* and *Unprotected*. Even within the *Mara Reserve,* cover < 20% is not a preferred habitat for elephants. These results suggest that human modification and habitat conversion in such areas would potentially have the least adverse effects on elephant space use in the ecosystem. Across management zones, avoidance of slope was the most consistent response, which is consistent with multiple other studies showing elephants do not like steep terrain [25, 36, 43, 85] and supporting hypothesis 2.

Across other models, drivers of differentiation varied. For the seasonal models, which were the most consistent of any model contrast conducted, agricultural use was the strongest driver of differentiation. This is likely because agriculture is highly seasonal and its use differs substantially across individuals [83]. Within the diurnal contrast, avoidance of settlement was the strongest driver of differentiation. Elephants avoided settlements more strongly during the day when humans were active, providing further evidence of the importance of human activity in structuring resource selection and space use. Across sexes, use of drainages appeared to be the most different, suggesting that ecological differences, possibly related to foraging behavior, drove habitat selection differences. Avoidance of settlements was the most consistent, indicating the influence of humans on resource selection was similar regardless of sex.

### Implications for management and conservation

The intra-strata differences demonstrated notable variation in selection behavior in relation to different human activities. The variation reported here highlights the adaptability of elephants to co-occur with humans by changing selection behaviour from what is ecologically optimal behaviour within the Mara Reserve, to what is tolerable from a risk–benefit trade-off within the unprotected regions. However, we note that two human activities that are rapidly increasing in the GME – fencing and agriculture – are generally considered incompatible with healthy elephant ranging behaviour. The rise in agricultural practice within the GME is leading to increased incidents of human-elephant conflict (MEP unpublished data), while fencing has the potential to inhibit movement critical to the biology of the species.

Drainages form naturally long linear features supporting high canopy riparian cover and are therefore especially important features to consider both from a connectivity and optimal elephant habitat perspective. Across all strata contrasts, the importance of drainages and high canopy cover forests was notable. These resources are relatively rare in the ecosystem but appear to be the key driver of space use and particularly important in unprotected areas. Maintaining elephant access to these resources in unprotected areas in the face of fencing and livestock pressures should be a priority for management planning. These areas are also preferred sites for lodges and tourism infrastructure, which are generally electrically fenced leading to total exclusion of elephants. Management planning focused on elephants should focus on reducing isolation, conversion and exclusionary developments within these forest patches, particularly as tourism continues to expand in the Mara. Given the lower selection for open grasslands, focusing future development in these areas could benefit elephants.

While we focused on the highest density of use for our resource selection analysis, it is notable that the study elephants were wide ranging with cross border excursions into Tanzania and movements into the Loita and Nyekweri forests, both of which are under threat (Fig. 1). Historically, connectivity between the Mau forest and the Mara may have occurred frequently, but we did not find evidence of this connection over 11 years of movement tracking. Maintaining movement connectivity across the ecosystem should be a core focus of management efforts going forward.

### Future directions

This study represents a comprehensive analysis of elephant resource selection across 11 years, without assessing dynamics within or across years. During the study period, habitat change occurred, namely in the form of agriculture and fencing expansion. Additionally, it has been shown that there is high individual variation in elephant populations in regards to resource selection particularly in response to human development [85]. While we accounted for individual variation in our hierarchical modeling approach, further work should explore how elephant space use and resource selection is structured by agricultural expansion in the context of seasonal dynamics and individual variation. Future work could also focus on identifying elephant movement corridors across the landscape given ongoing human development and land use change.

While we found seasonal dynamics to have minor influence on resource selection behavior, it is possible other inter-annual periods may be of importance in structuring elephant space use. In particular, the study system experiences major fluxes of biomass from the wildebeest and zebra migration [86, 87] that potentially strongly influence distributions of year-round resident species like elephants. Multi-species analyses to understand the impacts and interactions between species would be a valuable next step. Finally, the approach used here to contrast selection preference across different management regimes is readily translatable to other systems. Although management strategies are often specific to local geographic context, we hope that our approach of analysis of the differentiation in resource selection across management strategies will lead to insight into elephant spatial behaviour in other regions.

### Supplementary Information

**Additional file 1**. Supplementatry figures 1-6, tables 1-2 and details of the landcover classification.

**Additional file 2**. Analysis code.

**Additional file 3**. Figure of the number of monthly locations from each of the 49 tracking datasets for datasets included in the analysis.

## Data Availability

We provide access to the analysis notebooks and non-sensitive data used in our study in Additional File 2 and ask that requests for GPS tracking data (withheld for security reasons) be made to the corresponding author.

## Abbreviations

GPS: Global Positioning System
RSF: Resource Selection Function
NDVI: Normalized Difference Vegetation Index
GME: Greater Mara Ecosystem
IUCN: International Union for Conservation of Nature

## References

[1] Ceballos G, Ehrlich PR, Barnosky AD, García A, Pringle RM, Palmer TM (2015). Accelerated modern human–induced species losses: Entering the sixth mass extinction. Sci Adv.

[2] Haddad NM, Brudvig LA, Clobert J, Davies KF, Gonzalez A, Holt RD (2015). Habitat fragmentation and its lasting impact on Earth’s ecosystems. Sci Adv.

[3] Powers RP, Jetz W (2019). Global habitat loss and extinction risk of terrestrial vertebrates under future land-use-change scenarios. Nat Clim Chang.

[4] Johnson CJ, Seip DR, Boyce MS (2004). A quantitative approach to conservation planning: using resource selection functions to map the distribution of mountain caribou at multiple spatial scales. J Appl Ecol.

[5] Graham M, Douglas-Hamilton I, Adams WM, Lee PC (2009). The movement of African elephants in a human-dominated land-use Mosaic. Anim Conserv.

[6] Morris DW (2003). Toward an ecological synthesis: a case for habitat selection. Oecologia.

[7] Rosenzweig ML (1981). A theory of habitat selection. Ecology.

[8] Schoener TW (1971). Theory of feeding strategies. Annu Rev Ecol Syst.

[9] Johnson DH (1980). The comparison of usage and availability measurements for evaluating resource preference. Ecology.

[10] Gaynor KM, Hojnowski CE, Carter NH, Brashares JS (2018). The influence of human disturbance on wildlife nocturnality. Science.

[11] Brown JS, Laundré JW, Gurung M (1999). The ecology of fear: optimal foraging, game theory, and trophic interactions. J Mammal.

[12] Christie KS, Jensen WF, Boyce MS (2017). Pronghorn resource selection and habitat fragmentation in North Dakota. J Wildl Manag.

[13] Clinchy M, Zanette LY, Roberts D, Suraci JP, Buesching CD, Newman C (2016). Fear of the human “super predator” far exceeds the fear of large carnivores in a model mesocarnivore. Behav Ecol.

[14] Dill LM, Frid A (2020). Behaviourally mediated biases in transect surveys: a predation risk sensitivity approach. Can J Zool.

[15] Cleveland SM, Hebblewhite M, Thompson M, Henderson R (2012). Linking Elk movement and resource selection to hunting pressure in a heterogeneous landscape. Wildl Soc Bull.

[16] Branco PS, Merkle JA, Pringle RM, Pansu J, Potter AB, Reynolds A (2019). Determinants of elephant foraging behaviour in a coupled human-natural system: Is brown the new green?. J Anim Ecol.

[17] Thurfjell H, Spong G, Ericsson G (2013). Effects of hunting on wild boar Sus scrofa behaviour. Wildl Biol.

[18] Sih A, Ferrari MCO, Harris DJ (2011). Evolution and behavioural responses to human-induced rapid environmental change. Evol Appl.

[19] Boyce MS, McDonald LL (1999). Relating populations to habitats using resource selection functions. Trends Ecol Evol.

[20] Manly BFJ, McDonald LL, Thomas DL, McDonald TL, Erickson WP (2002). Resource selection by animals: statistical design and analysis for field studies.

[21] Boyce MS, Vernier PR, Nielsen SE, Schmiegelow FKA (2002). Evaluating resource selection functions. Ecol Model.

[22] Johnson CJ, Nielsen SE, Merrill EH, McDonald TL, Boyce MS (2006). Resource selection functions based on use-availability data: theoretical motivation and evaluation methods. J Wildl Manag.

[23] Hebblewhite M, Merrill E, McDermid G (2008). A multi-scale test of the forage maturation hypothesis in a partially migratory ungulate population. Ecol monogr.

[24] Newsome TM, Ballard G-A, Dickman CR, Fleming PJS, van de Ven R (2013). Home range, activity and sociality of a top predator, the dingo: a test of the Resource Dispersion Hypothesis. Ecography.

[25] Roever CL, van Aarde RJ, Leggett K (2012). Functional responses in the habitat selection of a generalist mega-herbivore, the African savannah elephant. Ecography.

[26] Bright Ross JG, Peters W, Ossi F, Moorcroft PR, Cordano E, Eccel E (2021). Climate change and anthropogenic food manipulation interact in shifting the distribution of a large herbivore at its altitudinal range limit. Sci Rep.

[27] Osipova L, Okello MM, Njumbi SJ, Ngene S, Western D, Hayward MW (2019). Using step-selection functions to model landscape connectivity for African elephants: accounting for variability across individuals and seasons. Anim Conserv.

[28] Zeller KA, McGarigal K, Cushman SA, Beier P, Vickers TW, Boyce WM (2017). Sensitivity of resource selection and connectivity models to landscape definition. Landscape Ecol.

[29] Stabach JA, Wittemyer G, Boone RB, Reid RS, Worden JS (2016). Variation in habitat selection by white-bearded wildebeest across different degrees of human disturbance. Ecosphere.

[30] Mysterud A, Ims RA (1998). Functional responses in habitat use: availability influences relative use in trade-off situations. Ecology.

[31] Hebblewhite M, Merrill E (2008). Modelling wildlife–human relationships for social species with mixed-effects resource selection models. J Appl Ecol.

[32] Moreau G, Fortin D, Couturier S, Duchesne T (2012). Multi-level functional responses for wildlife conservation: the case of threatened caribou in managed boreal forests. J Appl Ecol.

[33] Leclerc M, Vander Wal E, Zedrosser A, Swenson JE, Kindberg J, Pelletier F (2016). Quantifying consistent individual differences in habitat selection. Oecologia.

[34] Harju SM, Dzialak MR, Osborn RG, Hayden-Wing LD, Winstead JB (2011). Conservation planning using resource selection models: altered selection in the presence of human activity changes spatial prediction of resource use. Anim Conserv.

[35] Bastille-Rousseau G, Wall J, Douglas-Hamilton I, Lesowapir B, Loloju B, Mwangi N (2019). Landscape-scale habitat response of African elephants shows strong selection for foraging opportunities in a human dominated ecosystem. Ecography.

[36] Wall J, Wittemyer G, Klinkenberg B, LeMay V, Blake S, Strindberg S (2021). Human footprint and protected areas shape elephant range across Africa. Current Biol.

[37] Western D, Russell S, Cuthill I (2009). The status of wildlife in protected areas compared to non-protected areas of Kenya. PLoS ONE.

[38] Gobush K, Edwards C, Balfour D, Wittemyer G, Maisels F, Taylor R. Loxodonta africana. The IUCN Red List of Threatened Species 2021: e.T181008073A181022663. [Internet]. 2021 Apr. Available from: 10.2305/IUCN.UK.2021-1.RLTS.T181008073A181022663.en.

[39] Harris GM, Russell GJ, van Aarde RI, Pimm SL (2008). Rules of habitat use by elephants Loxodonta africana in southern Africa: insights for regional management. Oryx.

[40] Gara TW, Wang T, Skidmore AK, Zengeya FM, Ngene SM, Murwira A (2017). Understanding the effect of landscape fragmentation and vegetation productivity on elephant habitat utilization in Amboseli ecosystem Kenya. Afr J Ecol.

[41] de Knegt HJ, van Langevelde F, Skidmore AK, Delsink A, Slotow R, Henley S (2011). The spatial scaling of habitat selection by African elephants. J Anim Ecol.

[42] Loarie SR, Aarde RJV, Pimm SL (2009). Fences and artificial water affect African savannah elephant movement patterns. Biol Cons.

[43] Wall J, Douglas-Hamilton I, Vollrath F (2006). Elephants avoid costly mountaineering. Curr Biol.

[44] Galvin KA, Beeton TA, Luizza MW (2018). African community-based conservation: a systematic review of social and ecological outcomes. Ecol Soc.

[45] Nelson F, Muyamwa-Mupeta P, Muyengwa S, Sulle E, Kaelo D (2021). Progress or regression? Institutional evolutions of community-based conservation in eastern and southern Africa. Conserv Sci Pract.

[46] Tsalyuk M, Kilian W, Reineking B, Getz WM (2019). Temporal variation in resource selection of African elephants follows long-term variability in resource availability. Ecol Monogr.

[47] Bedelian C, Ogutu JO (2017). Trade-offs for climate-resilient pastoral livelihoods in wildlife conservancies in the Mara ecosystem. Kenya Pastoralism.

[48] MMWCA. Maasai Mara Wildlife Conservancies Strategic Plan 2021–2025 [Internet]. Maasai Mara Wildlife Conservancies Association; 2021 p. 24. Available from: https://maraconservancies.org/wp-content/uploads/2022/07/MMWCA-SP-2021-2025-Abridged.pdf

[49] Mukeka JM, Ogutu JO, Kanga E, Røskaft E (2019). Human-wildlife conflicts and their correlates in Narok County Kenya. Global Ecol Conserv.

[50] Ogutu JO, Piepho H-P, Said MY, Ojwang GO, Njino LW, Kifugo SC (2016). Extreme Wildlife declines and concurrent increase in livestock numbers in Kenya: what are the causes?. PLoS ONE.

[51] Bartzke GS, Ogutu JO, Mukhopadhyay S, Mtui D, Dublin HT, Piepho H-P (2018). Rainfall trends and variation in the Maasai Mara ecosystem and their implications for animal population and biodiversity dynamics. PLoS ONE.

[52] Nkedianye D, de Leeuw J, Ogutu JO, Said MY, Saidimu TL, Kifugo SC (2011). Mobility and livestock mortality in communally used pastoral areas: the impact of the 2005–2006 drought on livestock mortality in Maasailand. Pastoralism.

[53] Ogutu JO, Piepho HP, Dublin HT, Bhola N, Reid RS (2008). Rainfall influences on ungulate population abundance in the Mara-Serengeti ecosystem. J Anim Ecol.

[54] Kenya Wildlife Service. KWS NATIONAL WILDLIFE CENSUS 2021 REPORT [Internet]. 2021 May. Available from: https://kws.go.ke/content/national-wildlife-census-2021-report

[55] Løvschal M, Juul Nørmark M, Svenning J-C, Wall J (2022). New land tenure fences are still cropping up in the Greater Mara. Sci Rep.

[56] Mundia C, Murayama Y (2009). Analysis of land use/cover changes and animal population dynamics in a wildlife sanctuary in East Africa. Remote Sensing.

[57] Moss CJ. Getting to know a population. Studying Elephants. Nairobi, Kenya: African Wildlife Foundation; 1996. p. 58–74.

[58] Wall J, Wittemyer G, LeMay V, Douglas-Hamilton I, Klinkenberg B (2014). Elliptical time-density model to estimate wildlife utilization distributions. Methods Ecol Evol.

[59] Northrup JM, Hooten MB, Anderson CRJr, Wittemyer G. (2013). Practical guidance on characterizing availability in resource selection functions under a use – availability design. Ecology.

[60] Farr TG, Rosen PA, Caro E, Crippen R, Duren R, Hensley S, et al. The Shuttle Radar Topography Mission. Reviews of Geophysics [Internet]. 2007 [cited 2023 Jun 23];45. Available from: https://onlinelibrary.wiley.com/doi/abs/10.1029/2005RG000183

[61] Didan K. MOD13Q1 MODIS/Terra vegetation indices 16-day L3 global 250m SIN grid V006. NASA EOSDIS Land Processes DAAC. 2015;10.

[62] Grill G, Lehner B, Thieme M, Geenen B, Tickner D, Antonelli F (2019). Mapping the world’s free-flowing rivers. Nature.

[63] Li W, Buitenwerf R, Munk M, Bøcher PK, Svenning J-C (2020). Deep-learning based high-resolution mapping shows woody vegetation densification in greater Maasai Mara ecosystem. Remote Sens Environ.

[64] Schielzeth H (2010). Simple means to improve the interpretability of regression coefficients. Methods Ecol Evol.

[65] Zuur AF, Ieno EN, Saveliev AA. Beginner’s Guide to Spatial, Temporal, and Spatial-Temporal Ecological Data Analysis with R-INLA. Volume I: Using GLM and GLMM. Newburgh, United Kingdom: Highland Statistics Ltd.; 2017.

[66] Bastille-Rousseau G, Wittemyer G (2022). Simple metrics to characterize inter-individual and temporal variation in habitat selection behaviour. J Anim Ecol.

[67] Tyrrell P, Amoke I, Betjes K, Broekhuis F, Buitenwerf R, Carroll S (2022). Landscape Dynamics (landDX) an open-access spatial-temporal database for the Kenya-Tanzania borderlands. Sci Data.

[68] Gorelick N, Hancher M, Dixon M, Ilyushchenko S, Thau D, Moore R (2017). Google Earth Engine: planetary-scale geospatial analysis for everyone. Remote Sens Environ.

[69] Rue H, Martino S, Chopin N (2009). Approximate Bayesian inference for latent Gaussian models by using integrated nested Laplace approximations. J Royal Stat Soc.

[70] van de Water A, Henley M, Bates L, Slotow R (2022). The value of elephants: A pluralist approach. Ecosyst Serv.

[71] Hoare R (2015). Lessons from 20 years of human-elephant conflict mitigation in Africa. Hum Dimens Wildl.

[72] Martínez-Freiría F, Tarroso P, Rebelo H, Brito JC (2016). Contemporary niche contraction affects climate change predictions for elephants and giraffes. Divers Distrib.

[73] Schlossberg S, Chase MJ, Gobush KS, Wasser SK, Lindsay K (2020). State-space models reveal a continuing elephant poaching problem in most of Africa. Sci Rep.

[74] Wittemyer G, Northrup JM, Blanc J, Douglas-Hamilton I, Omondi P, Burnham KP (2014). Illegal killing for ivory drives global decline in African elephants. Proc Natl Acad Sci.

[75] Løvschal M, Bøcher PK, Pilgaard J, Amoke I, Odingo A, Thuo A (2017). Fencing bodes a rapid collapse of the unique Greater Mara ecosystem. Sci Rep.

[76] Tyrrell P, Buitenwerf R, Brehony P, Løvschal M, Wall J, Russell S, et al. Wide-scale subdivision and fencing of southern Kenyan rangelands jeopardizes biodiversity conservation and pastoral livelihoods: Demonstration of utility of open-access landDX database. Frontiers in Conservation Science [Internet]. 2022 [cited 2023 Jun 23];3. Available from: https://www.frontiersin.org/articles/10.3389/fcosc.2022.889501

[77] Veldhuis MP, Ritchie ME, Ogutu JO, Morrison TA, Beale CM, Estes AB (2019). Cross-boundary human impacts compromise the Serengeti-Mara ecosystem. Science.

[78] Kweyu RM, Thenya T, Kiemo K, Emborg J (2020). The nexus between land cover changes, politics and conflict in Eastern Mau forest complex Kenya. Appl Geogr.

[79] Mukeka JM, Ogutu JO, Kanga E, Roskaft E (2018). Characteristics of Human-Wildlife Conflicts in Kenya: examples of Tsavo and Maasai Mara Regions. Environ Nat Resour Res.

[80] Walpole MJ, Leader-Williams N (2001). Masai Mara tourism reveals partnership benefits. Nature.

[81] de Boer WF, van Langevelde F, Prins HHT, de Ruiter PC, Blanc J, Vis MJP (2013). Understanding spatial differences in African elephant densities and occurrence, a continent-wide analysis. Biol Conserv.

[82] Wall J, Wittemyer G, Klinkenberg B, LeMay V, Douglas-Hamilton I (2013). Characterizing properties and drivers of long distance movements by elephants (Loxodonta africana) in the Gourma Mali. Biol Conserv.

[83] Hahn NR, Wall J, Denninger-Snyder K, Goss M, Sairowua W, Mbise N (2022). Risk perception and tolerance shape variation in agricultural use for a transboundary elephant population. J Anim Ecol.

[84] Ihwagi FW, Thouless C, Wang T, Skidmore AK, Omondi P, Douglas-Hamilton I (2018). Night-day speed ratio of elephants as indicator of poaching levels. Ecol Ind.

[85] Bastille-Rousseau G, Wittemyer G (2019). Leveraging multidimensional heterogeneity in resource selection to define movement tactics of animals. Ecol Lett.

[86] Holdo RM, Holt RD, Coughenour MB, Ritchie ME (2007). Plant productivity and soil nitrogen as a function of grazing, migration and fire in an African savanna. J Ecol.

[87] McNaughton SJ (1976). Serengeti Migratory Wildebeest: Facilitation of Energy Flow by Grazing. Science.

