# Supplementary material for: Land use drives differential resource selection by African elephants in the Greater Mara Ecosystem, Kenya
Source: Mov Ecol. 2024 Feb 1;12:11. doi: 10.1186/s40462-023-00436-8 (PMC10832223; doi:10.1186/s40462-023-00436-8)
Supplement: Supplementary file 1 — Additional file 1. Supplementatry figures 1-6, tables 1-2 and details of the landcover classification. [file 40462_2023_436_MOESM1_ESM.docx]

# Supplementary Information

## SM: Figures & Tables


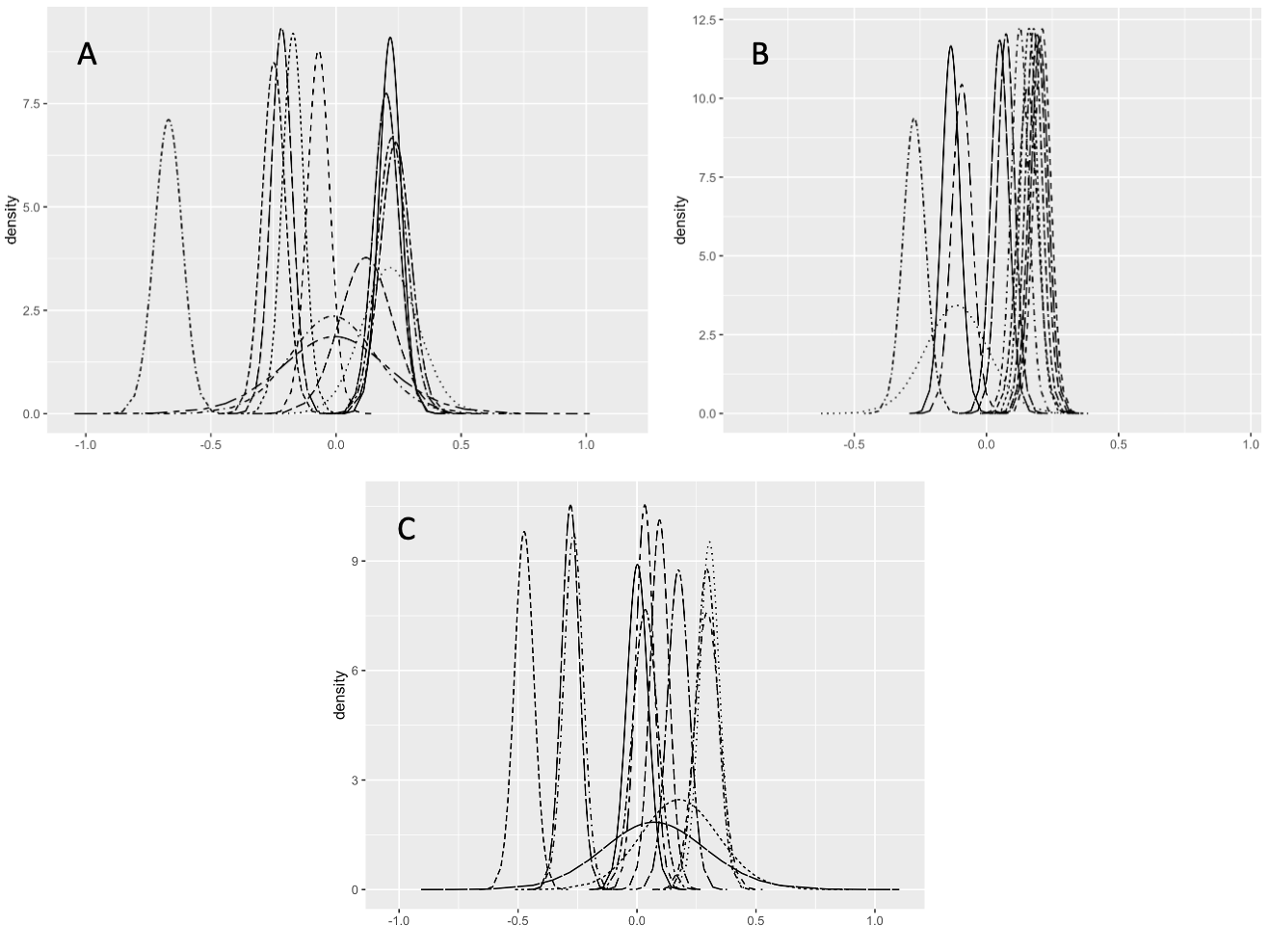


# SM Figure 1. Distribution of the random effects from individual elephants in each zonal model. A) is the Mara Reserve (Mara Reserve), B) is the community conservancy areas (Conservancies) zone, and C) is the unprotected area (Unprotected). Visual inspection of the distributions and the standard deviation of the hyperparameters show that there is more variation between individuals in the nationally protected and the unprotected zones, while the variation is smaller in the community conservancies.

#


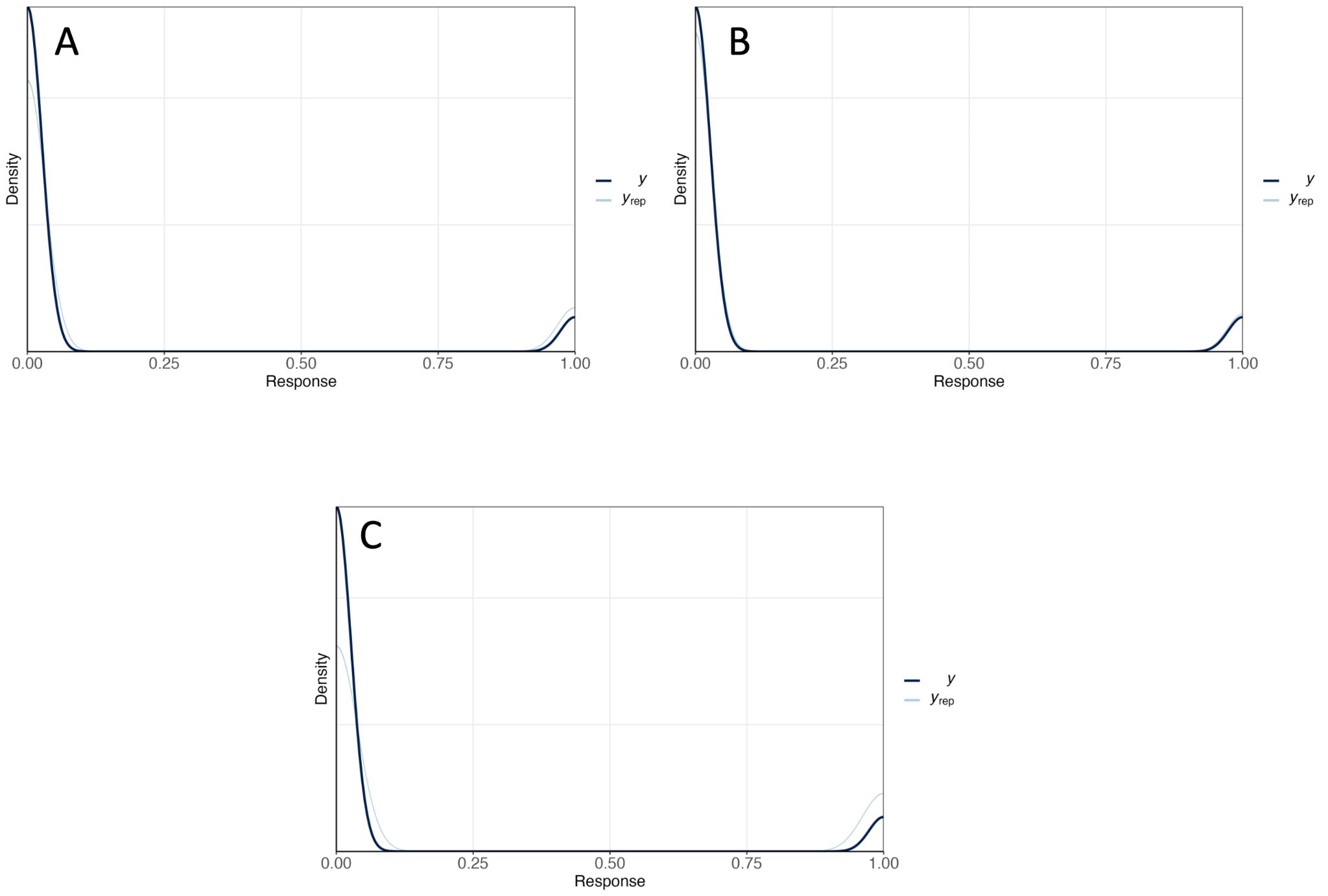


# SM Figure 2. Posterior predictive checks of each zonal model showed a similar distribution of the observed and predicted values, indicating that the models accurately described the data. Plots are shown for the A) Mara Reserve , B) Conservancies, and C) Unprotected models. The black line represents the observed distribution of used (elephant GPS relocations) and unused (random GPS relocations) data, and the blue lines represent the distribution of predicted used and unused relocations based on 500 replications.


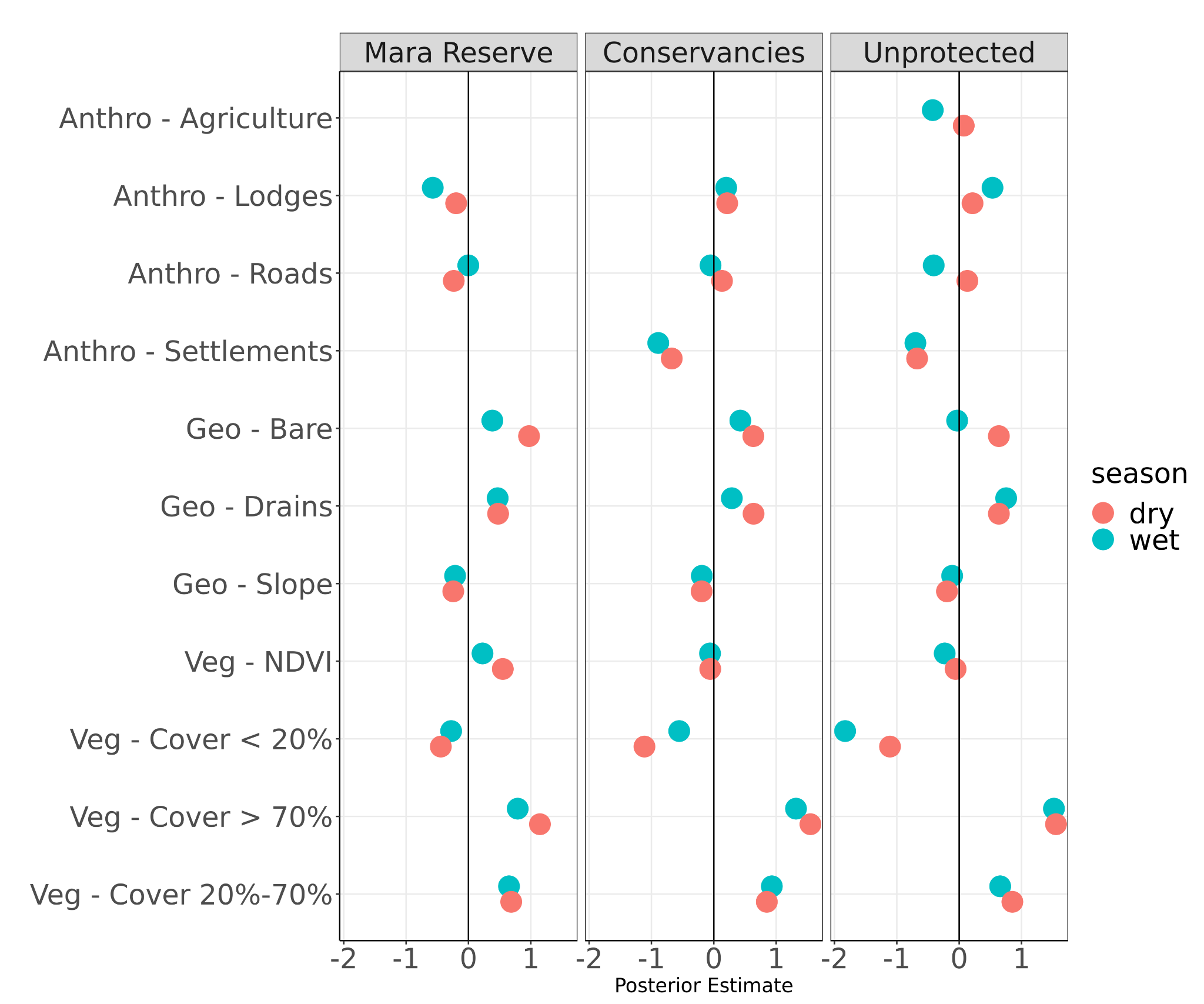


**SM Figure 3**. Mean posterior and 95% highest-density posterior intervals (HDPI) (x-axis) for each covariate (y-axis) within the Seasonal set of models.

#
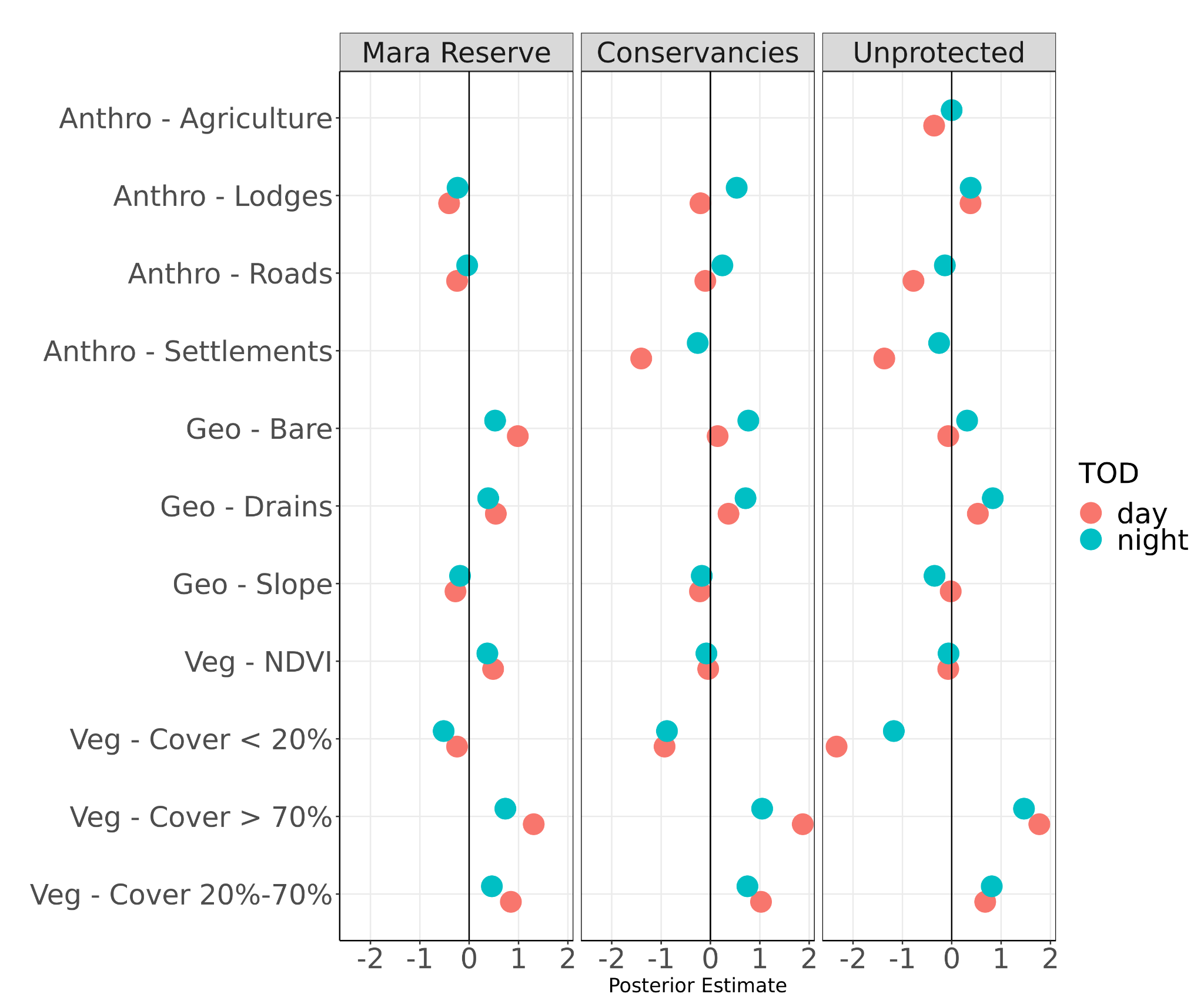


# SM Figure 4. Mean posterior and 95% highest-density posterior intervals (HDPI) (x-axis) for each covariate (y-axis) within the Time-of-Day set of models.

#


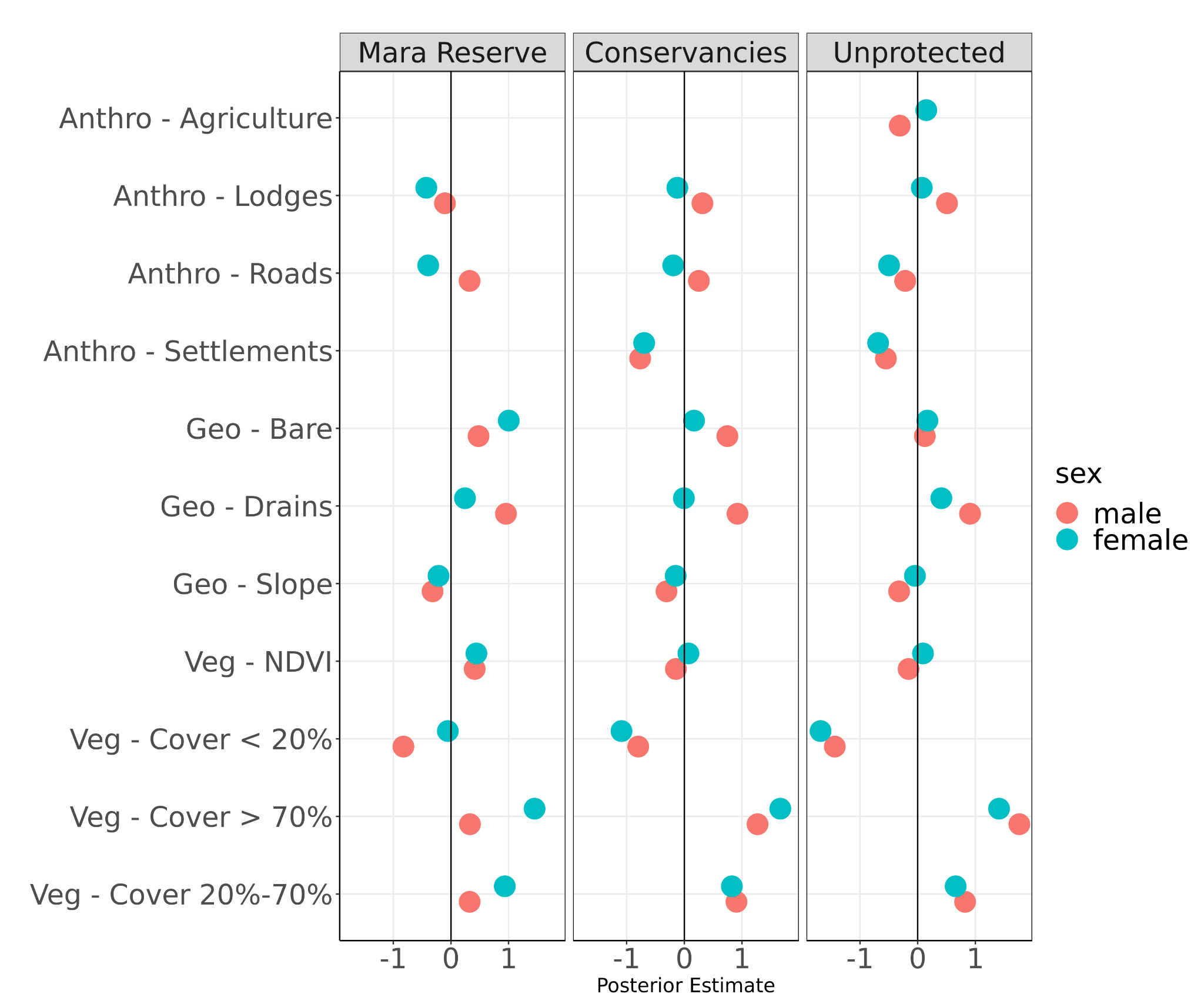


**SM Figure 5**. Mean posterior and 95% highest-density posterior intervals (HDPI) (x-axis) for each covariate (y-axis) within the Sex set of models.

#
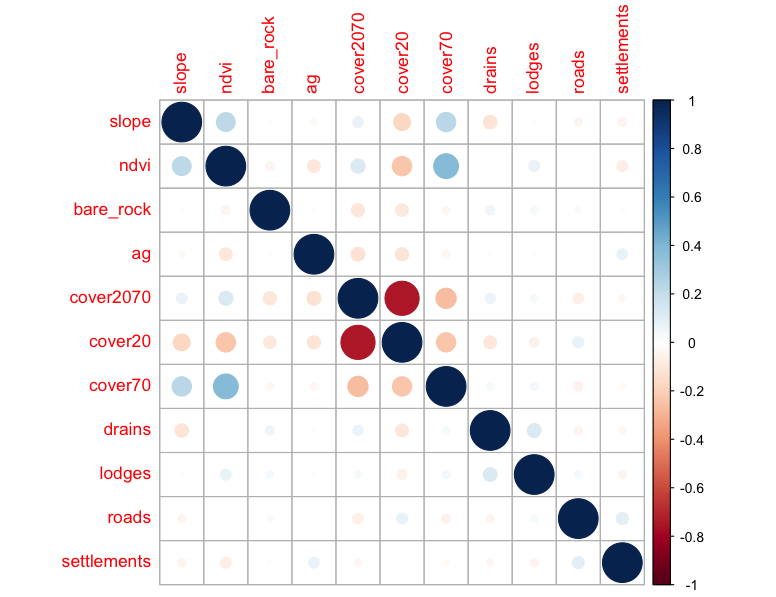


**SM Figure 6.** Correlation of covariates used in the resource selection models.

<Additional File 3>

**SM Figure 7:** The number of monthly locations from each of the 49 tracking datasets for datasets included in the analysis. Collars were most commonly set to record 1 GPS fix per hour.

#

# Table SM 1. Model coefficient estimates and 95% Highest-Density Posterior Interval (HDPI) for the Zone-only RSF model.

| **Coefficient** | **Estimate** | **HDPI Lower** | **HDPI Upper** | **Zone** |
| --- | --- | --- | --- | --- |
| NDVI | 0.430 | 0.420 | 0.441 | Mara Reserve |
| Slope | -0.232 | -0.239 | -0.225 | Mara Reserve |
| Veg.Cover < 20% | -0.399 | -0.490 | -0.308 | Mara Reserve |
| Veg.Cover 20%-70% | 0.636 | 0.545 | 0.727 | Mara Reserve |
| Veg.Cover > 70% | 1.014 | 0.920 | 1.108 | Mara Reserve |
| Bare Rock | 0.741 | 0.639 | 0.843 | Mara Reserve |
| Drains | 0.469 | 0.457 | 0.482 | Mara Reserve |
| Lodges | -0.328 | -0.369 | -0.286 | Mara Reserve |
| Roads | -0.144 | -0.159 | -0.128 | Mara Reserve |
| NDVI | -0.061 | -0.065 | -0.057 | Conservancies |
| Slope | -0.196 | -0.199 | -0.192 | Conservancies |
| Veg.Cover < 20% | -0.912 | -0.928 | -0.896 | Conservancies |
| Veg.Cover 20%-70% | 0.876 | 0.861 | 0.891 | Conservancies |
| Veg.Cover > 70% | 1.482 | 1.464 | 1.499 | Conservancies |
| Bare Rock | 0.587 | 0.560 | 0.615 | Conservancies |
| Drains | 0.531 | 0.524 | 0.538 | Conservancies |
| Lodges | 0.211 | 0.196 | 0.226 | Conservancies |
| Roads | 0.074 | 0.067 | 0.082 | Conservancies |
| Settlements | -0.738 | -0.747 | -0.729 | Conservancies |
| NDVI | -0.067 | -0.073 | -0.061 | Unprotected |
| Slope | -0.153 | -0.158 | -0.148 | Unprotected |
| Veg.Cover < 20% | -1.519 | -1.559 | -1.479 | Unprotected |
| Veg.Cover 20%-70% | 0.753 | 0.721 | 0.785 | Unprotected |
| Veg.Cover > 70% | 1.634 | 1.599 | 1.668 | Unprotected |
| Bare Rock | 0.161 | 0.113 | 0.209 | Unprotected |
| Agriculture | -0.131 | -0.170 | -0.092 | Unprotected |
| Drains | 0.708 | 0.697 | 0.719 | Unprotected |
| Lodges | 0.338 | 0.284 | 0.393 | Unprotected |
| Roads | -0.336 | -0.354 | -0.317 | Unprotected |
| Settlements | -0.607 | -0.621 | -0.593 | Unprotected |

**SM - Land Cover Classification Details**

The land cover classification was created using Sentinel-1 and Sentinel-2 satellite data with a Random Forest model on the Google Earth Engine platform.

*Data collection*

We collected 2,574 ground truth land cover data points and 122 remotely collected data points from 2019-2021, using the following classifications: (1) rock/bare/built, (2) agriculture, (3) greater than 70% cover (forest, thicket), (4) 20-70% cover (shrubland, open thicket, woodland), (5) less than 20% cover (wooded grassland and grassland). To provide spatial coverage in areas that were difficult to access we scored Sentinel-2 imagery in Google Earth Engine from 2020 and 2021.

*Satellite imagery*

We created a multi-year composite using Sentinel-1 and Sentinel-2 surface reflectance (SR) imagery for 2019-2021. Collectively, the European Space Agency (ESA) twin satellites, Sentinel-2A and Sentinel-2B, referred to as Sentinel-2, have a five day revisit period and 13 spectral bands from visible to shortwave infrared at 10-20 m resolution. Sentinel-2 SR has been processed with an atmospheric correction applied to Top-Of-Atmosphere (TOA) Level-1C orthoimage products. We used a cloud score of less than 30% per pixel to create a cloud mask. We then used the following bands from Sentinel-2: blue, green, red, red edge 1, red edge 2, red edge 3, red edge 4, near-infrared, short wave infrared 1, and shortwave infrared 2. We used VV and VH from Sentinel-1 ascending orbit.

Using these bands, we created the following indices: Enhanced Vegetation Index (EVI), green brown vegetation index (GBVI), Green chlorophyll vegetation index (GCVI), Green red vegetation index (GRVI), Normalized Difference Vegetation Index (NDVI), and Normalized Difference Moisture Index (NDMI). For each band and index we used the 10th percentile, 25th percentile, 50th percentile, 75th percentile, 90th percentile, the difference between the 90th and 10th percentiles, and the difference between the 75th and 25th percentiles for the study period. We also included slope, elevation, and aspect, from the Shuttle Radar Topography Mission (Farr et al. 2007) at a resolution of 1 arc-second (approximately 30m).

*Results*

**SM Table 2**. Accuracy metrics from 5-fold cross validation of the land cover classification. After excluding the degraded class which performed poorly, the mean accuracy across all classes was 81%.

| **Metric** | **Bare/Built** | **Crop** | **<20% Cover** | **20-70% Cover** | **>70% Cover** | **Degraded** |
| --- | --- | --- | --- | --- | --- | --- |
| Sensitivity | 0.75322 | 0.7198 | 0.7986 | 0.8366 | 0.79284 | 0.37124 |
| Specificity | 0.99097 | 0.9662 | 0.9512 | 0.8002 | 0.97626 | 0.98431 |
| Pos Pred Value | 0.92368 | 0.8384 | 0.7545 | 0.6748 | 0.79897 | 0.67683 |
| Neg Pred Value | 0.96511 | 0.9339 | 0.9618 | 0.9081 | 0.97536 | 0.94647 |
| Prevalence | 0.12677 | 0.1961 | 0.1581 | 0.3313 | 0.10637 | 0.08134 |
| Detection Rate | 0.09548 | 0.1412 | 0.1262 | 0.2772 | 0.08433 | 0.0302 |
| Detection Prevalence | 0.10337 | 0.1684 | 0.1673 | 0.4108 | 0.10555 | 0.04461 |
| Balanced Accuracy | 0.87209 | 0.843 | 0.8749 | 0.8184 | 0.88455 | 0.67777 |
