# Supplementary figures and images for: Land use drives differential resource selection by African elephants in the Greater Mara Ecosystem, Kenya
Source: Mov Ecol. 2024 Feb 1;12:11. doi: 10.1186/s40462-023-00436-8 (PMC10832223; doi:10.1186/s40462-023-00436-8)

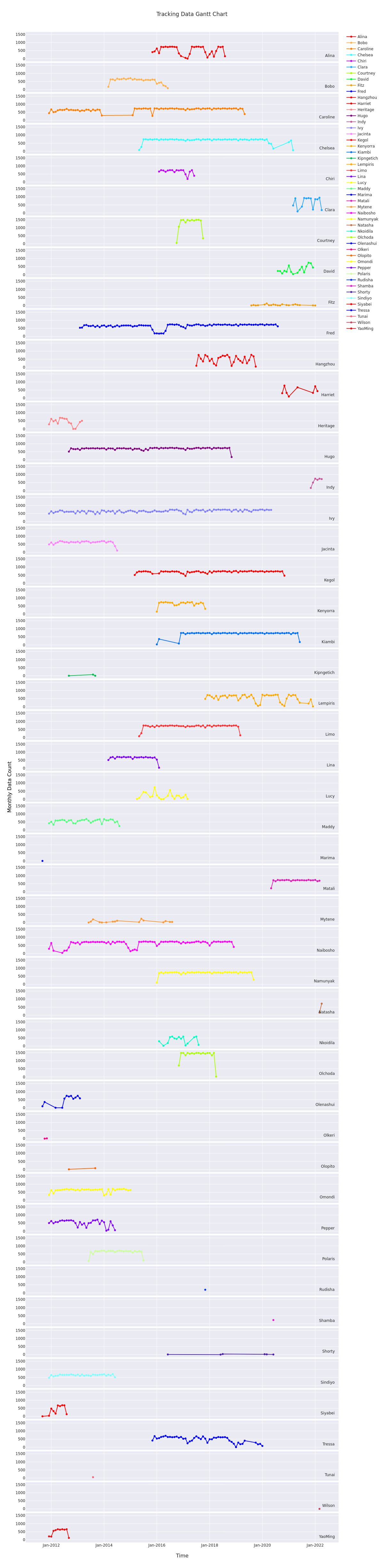

Supplement: Supplementary file 2 — Additional file 2. Analysis code. [file 40462_2023_436_MOESM2_ESM.zip › RSF Supp Info/workspace/notebooks/5 - RSF Analysis/tracking_data_gantt_chart.png]

Supplement: Supplementary file 2 — Additional file 2. Analysis code. [file 40462_2023_436_MOESM2_ESM.zip › RSF Supp Info/workspace/notebooks/3 - Spatial Data/rsf_roads_buffer400m.tif]

Supplement: Supplementary file 2 — Additional file 2. Analysis code. [file 40462_2023_436_MOESM2_ESM.zip › RSF Supp Info/workspace/notebooks/3 - Spatial Data/rsf_settlements_buffer400m.tif]

Supplement: Supplementary file 2 — Additional file 2. Analysis code. [file 40462_2023_436_MOESM2_ESM.zip › RSF Supp Info/workspace/notebooks/3 - Spatial Data/rsf_lodges_buffer400m.tif]

Supplement: Supplementary file 2 — Additional file 2. Analysis code. [file 40462_2023_436_MOESM2_ESM.zip › RSF Supp Info/workspace/notebooks/3 - Spatial Data/rsf_drains_buffer400m.tif]
